# Non-invasive CT radiomic biomarkers predict microsatellite stability status in colorectal cancer: a multicenter validation study

**DOI:** 10.1186/s41747-024-00484-8

**Published:** 2024-08-26

**Authors:** Zuhir Bodalal, Eun Kyoung Hong, Stefano Trebeschi, Ieva Kurilova, Federica Landolfi, Nino Bogveradze, Francesca Castagnoli, Giovanni Randon, Petur Snaebjornsson, Filippo Pietrantonio, Jeong Min Lee, Geerard Beets, Regina Beets-Tan

**Affiliations:** 1 Department of Radiology, The Netherlands Cancer Institute, Amsterdam, The Netherlands; 2 GROW Research Institute for Oncology and Developmental Biology, Maastricht University, Maastricht, The Netherlands; 3 Seoul National University Hospital, Seoul, South Korea; 4 Radiology Unit, Sant’Andrea Hospital, Sapienza University of Rome, Rome, Italy; 5 Department of Radiology, American Hospital Tbilisi, Tbilisi, Georgia; 6 Department of Radiology, Royal Marsden Hospital, London, UK; 7 Division of Radiotherapy and Imaging, The Institute of Cancer Research, London, UK; 8 Department of Medical Oncology, Fondazione IRCCS Istituto Nazionale dei Tumori di Milano, Milan, Italy; 9 Department of Pathology, Netherlands Cancer Institute, Amsterdam, The Netherlands; 10 Faculty of Medicine, University of Iceland, Reykjavik, Iceland; 11 Oncology and Hemato-oncology Department, University of Milan, Milan, Italy; 12 Department of Surgery, Netherlands Cancer Institute, Amsterdam, The Netherlands; 13 Institute of Regional Health Research, University of Southern Denmark, Odense, Denmark

**Keywords:** Colorectal neoplasms, DNA mismatch repair, Machine learning, Microsatellite instability, Radiomics

## Abstract

**Background:**

Microsatellite instability (MSI) status is a strong predictor of response to immunotherapy of colorectal cancer. Radiogenomic approaches promise the ability to gain insight into the underlying tumor biology using non-invasive routine clinical images. This study investigates the association between tumor morphology and the status of MSI *versus* microsatellite stability (MSS), validating a novel radiomic signature on an external multicenter cohort.

**Methods:**

Preoperative computed tomography scans with matched MSI status were retrospectively collected for 243 colorectal cancer patients from three hospitals: Seoul National University Hospital (SNUH);

Netherlands Cancer Institute (NKI); and Fondazione IRCCS Istituto Nazionale dei Tumori, Milan Italy (INT). Radiologists delineated primary tumors in each scan, from which radiomic features were extracted. Machine learning models trained on SNUH data to identify MSI tumors underwent external validation using NKI and INT images. Performances were compared in terms of area under the receiving operating curve (AUROC).

**Results:**

We identified a radiomic signature comprising seven radiomic features that were predictive of tumors with MSS or MSI (AUROC 0.69, 95% confidence interval [CI] 0.54−0.84, *p* = 0.018). Integrating radiomic and clinical data into an algorithm improved predictive performance to an AUROC of 0.78 (95% CI 0.60−0.91, *p* = 0.002) and enhanced the reliability of the predictions.

**Conclusion:**

Differences in the radiomic morphological phenotype between tumors MSS or MSI could be detected using radiogenomic approaches. Future research involving large-scale multicenter prospective studies that combine various diagnostic data is necessary to refine and validate more robust, potentially tumor-agnostic MSI radiogenomic models.

**Relevance statement:**

Noninvasive radiomic signatures derived from computed tomography scans can predict MSI in colorectal cancer, potentially augmenting traditional biopsy-based methods and enhancing personalized treatment strategies.

**Key Points:**

- Noninvasive CT-based radiomics predicted MSI in colorectal cancer, enhancing stratification.
- A seven-feature radiomic signature differentiated tumors with MSI from those with MSS in multicenter cohorts.
- Integrating radiomic and clinical data improved the algorithm’s predictive performance.

**Graphical Abstract:**

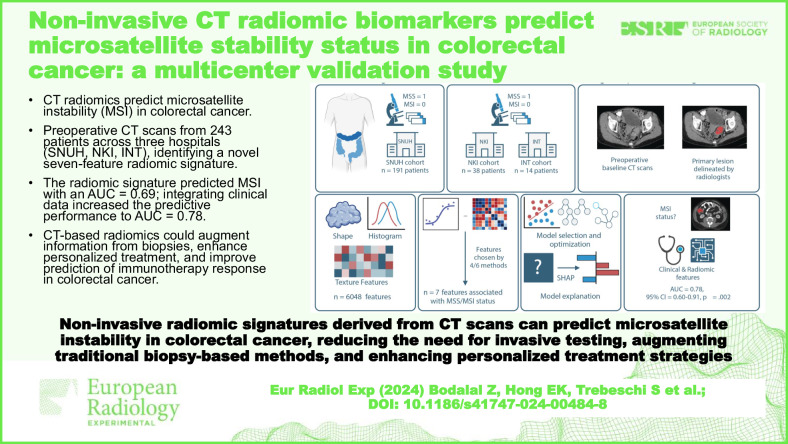

## Background

Microsatellite instability (MSI) is associated with a genomic hypermutable state that occurs due to defects in the DNA mismatch repair (MMR) mechanism. Cells with deficient MMR are unable to efficiently correct DNA replication errors, resulting in the accumulation of mutations [1]. In colorectal cancer (CRC), MSI is found in approximately 15% of cases [2, 3]. It has been established that there are differences between CRC tumors with microsatellite stability (MSS) and MSI in terms of molecular, histopathological, clinical, and treatment outcomes [4]. Microsatellite instability status is an important prognostic marker, particularly in stage II–III CRC, where MSI status confers a better prognosis than MSS [5–9]. Additionally, MSI status is also a significant predictive marker for both adjuvant and neoadjuvant immunotherapy response in CRC [10–13].

MSI tumors exhibit increased immune infiltration [14, 15], presumably due to T-cell receptor recognition of neoantigens produced by mutations [16, 17]. These characteristics make them good candidates for immunotherapy [18–21]. The United States Food and Drug Administration has granted unique tissue-agnostic approval for the use of pembrolizumab across all MSI-high tumors [22]. As a result, MSI testing is now recommended to guide first-line treatment choices, and immunotherapy is advised in patients with MSI-high metastatic CRC [23–26].

Radiogenomic approaches are envisioned as valuable complements to biopsies rather than their replacements. Particularly for conditions such as Lynch syndrome, which is associated with MSI in tumors of various locations, primarily the colorectum and endometrium, radiogenomics could provide critical non-invasive assessments [27]. For metastasized tumors with lesions located in hard-to-reach or risk-prone zones like the subdiaphragmatic and pelvic areas, radiogenomics could offer vital diagnostic information. Additionally, imaging markers may be particularly beneficial in situations where hospitals lack the resources to routinely assess MSI/MSS status, such as in developing economies. Furthermore, when patients present with two or more primary tumors with different MSS/MSI statuses, radiomics approaches may help differentiate the origin of each lesion.

Identifying and validating noninvasive radiomic markers for MSI holds promise not only for routine CRC cases but also for tumor types where determining MSI status is not typically part of the standard workup. A significant study involving 5,930 cancer exomes from The Cancer Genome Atlas−TCGA found that 14 out of 18 cancer types had MSI tumors [28]. In all the abovementioned scenarios, radiogenomic approaches might help flag cases for detailed molecular analysis when resources, circumstances, or guidelines do not permit routine testing. Several single-center studies have attempted to predict the MSI status of CRC patients based on diagnostic computed tomography (CT) imaging [4, 29]. However, there is a critical need for external validation to test the robustness of the identified signatures/predictive algorithms using data from other centers/scanners.

In this study, we aimed to explore the usefulness of radiomic features (either alone or combined with clinical parameters) to noninvasively predict the MSS *versus* MSI status in CRC. We validated this predictive signature and algorithm on an external dataset from two independent centers to assess robustness and generalizability.

## Methods

### Patient consent, cohorts, and clinical data

All centers approved the use of data for this project, and the Institutional Review Board of the Netherlands Cancer Institute granted ethical clearance under the protocol number (IRBd19-147). This retrospective study used data derived from three oncological centers located in different countries: Netherlands Cancer Institute (NKI) in Amsterdam, The Netherlands; Seoul National University Hospital (SNUH) in Seoul, South Korea; and Fondazione IRCCS Istituto Nazionale dei Tumori (INT) in Milan, Italy.

**Fig. 1:**
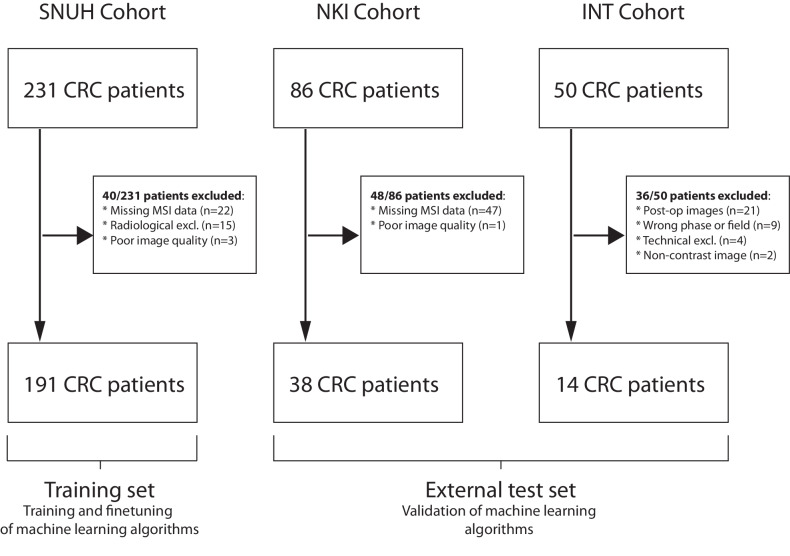
This schematic outlines how exclusion criteria were applied to the institutional database search results to form the datasets used in our analysis. “Radiological exclusion” includes reasons like the availability of only unenhanced images, inappropriate scan phases, and difficulties in tumor segmentation. MSI, Microsatellite instability; NKI, Netherlands Cancer Institute; SNUH, Seoul National University Hospital

**Fig. 2:**
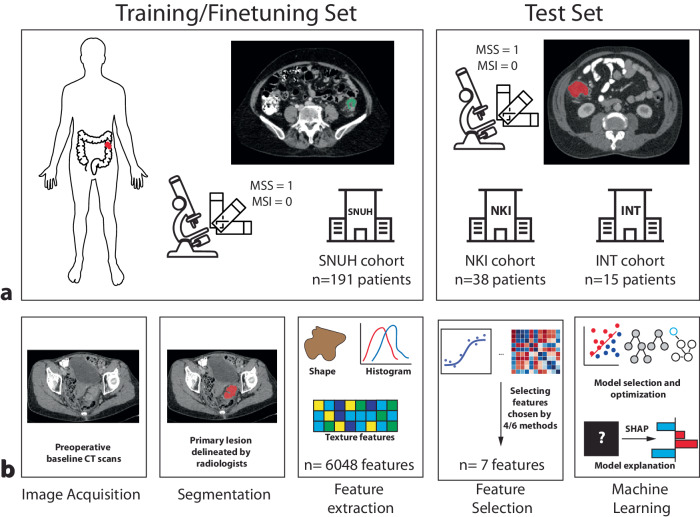
Study design and workflow. **a** Preoperative scans and corresponding MSI/MSS status data were collected from three centers. Data from SNUH were used as the training (and fine-tuning) cohort, while data from NKI and INT were combined to create the external validation set. **b** The radiomic workflow involved segmenting the primary tumor and extracting radiomic features. Supervised ensemble feature selection was followed by using a tree-based evolutionary algorithm to identify the optimal model for predicting MSS status. Shapley value analysis was employed to elucidate feature significance and address the artificial intelligence ‘black box’ issue. MSI, Microsatellite instability; MSS, Microsatellite stability; NKI, Netherlands Cancer Institute; SNUH, Seoul National University Hospital

An institutional database search was conducted to include all histologically proven CRC patients undergoing surgical tumor resection at the NKI from January 2010 to December 2017 and at the SNUH from January 2016 to December 2017, ensuring a comprehensive dataset that reflects real-world clinical outcomes over these periods. For the INT cohort, spanning June 2014 to January 2020, a random subsample of 50 patients was selected due to data processing constraints and the high volume of eligible cases. This sampling method was intended to maintain manageability and statistical randomness, preventing selection bias and allowing for meaningful data analysis. In total, 367 patients were identified as potential candidates, and their relevant preoperative contrast-enhanced CT images were retrieved from the Picture Archiving and Communication System. Figure 1 provides a comprehensive overview of the inclusion and exclusion of patients in the study. For every patient included in the final cohort, information on the MSI/MSS status (MSS = 0, MSI = 1), age, gender, and tumor location (right/left colon) were collected from their electronic health records. This study was designed to align with the CheckList for EvaluAtion of Radiomics research −CLEAR guidelines [30]. Figure 2 outlines the design and workflow of this study.

### Microsatellite stability status

The MSI/MSS status for all patients in our final cohort was determined using archival tissue. We obtained the status from the patients’ pathology reports, which were determined using standard-of-care procedures. The MSI/MSS status of the patients was identified through the analysis of formalin-fixed paraffin-embedded tissue samples obtained from tumor samples primarily gathered during endoscopic diagnostic biopsy procedures or surgical resection of the primary tumor. The samples were processed according to standard histopathological procedures. Following the fixation and embedding, representative sections were cut from the formalin-fixed paraffin-embedded blocks and reviewed by board-certified pathologists. Per institutional guidelines, MSI/MSS status was determined using immunohistochemistry and/or polymerase chain reaction methods.

Immunohistochemistry was used to assess the expression of mismatch repair (MMR) proteins, *i.e*., MLH1, MSH2, MSH6, and PMS2. The absence of nuclear staining in tumor cells of any of these proteins was classified as representing MSI. Polymerase chain reaction-based analysis was performed on selected cases, targeting specific microsatellite loci to confirm MSI or determine MSS status. While varying, these methodologies reflect current clinical practices and would provide a diverse enough sample to train a robust, generalizable model. Using standard-of-care procedures for MSI/MSS status determination ensured that our study was based on accurate and reliable data, minimizing the risk of misclassification.

### Image acquisition and segmentation

Patients were instructed to abstain from oral consumption 2–4 h before the CT scan, and no bowel preparation was conducted before the CT image acquisition. Scans were performed on GE Healthcare, SiemensHealthineers, Philips Healthcare, or Canon Medical Systems CT scanners with 16–320 channels. CT scans were reconstructed to 1–3 mm slice thickness. During the scan, patients were intravenously administered with iodine-based contrast agent (90–130 mL of Omnipaque at 300 mg/mL), and portovenous phase scans were acquired 70 s after administration, which we used in our image analysis. The in-plane resolution was consistent across the three imaging centers, indicating comparable diagnostic quality. At the NKI, the median *x* and *y* pixel spacings were 0.763 mm (interquartile range [IQR] 0.691–0.794 mm). INT and SNUH both showed similar in-plane resolutions with median spacings of 0.705 mm (IQR (0.645–0.775 mm)) and 0.684 mm (IQR (0.648–0.715 mm)), respectively.

**Fig. 3:**
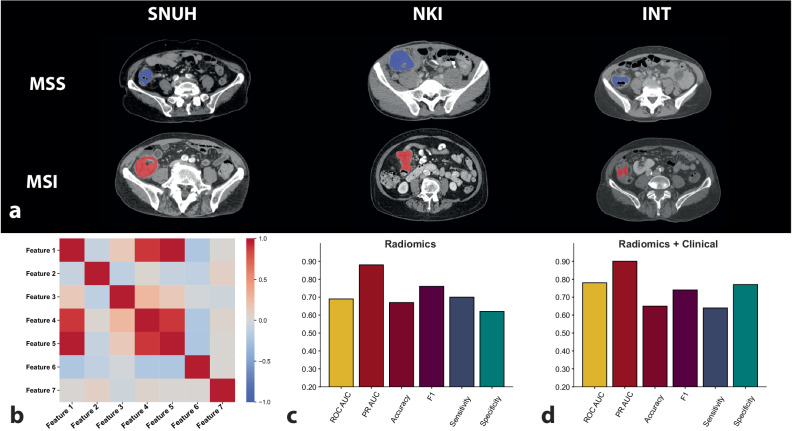
Performance evaluation. **a** Representative examples of primary tumor segmentation in MSS and MSI patients from all three cohorts. **b** Correlation matrix depicting relationships among features in the radiomic signature. **c**, **d** Comparative analysis of predictive performance on the external validation set for models trained exclusively on radiomics data *versus* those trained on integrated radiomic and clinical data. MSI, Microsatellite instability; MSS, Microsatellite stability; NKI, Netherlands Cancer Institute; SNUH, Seoul National University Hospital

The contrast-enhanced CT images were transferred from the Picture Archiving and Communication System to a local workstation for image segmentation and analysis. A team of radiologists manually delineated primary tumors on the preoperative CT scans (E.K.H., N.B., F.L., and F.C., with 6, 6, 4, and 4 years of experience in abdominal radiology). The readers were blinded to all clinical and histopathological information apart from the tumor’s location. One radiologist (E.K.H.) conducted a quality check of all the segmentations to ensure uniformity. A dedicated image analysis program (3D Slicer, v.4.10.2, San Francisco, USA) was used to delineate/segment the whole primary tumor volumetrically in each of the slices of the axial portal venous images (Fig. 3a). All imaging and segmentation files were stored according to the Nearly Raw Raster Data−NRRD standard [31].

### Feature extraction and selection

From each segmented lesion, 6,048 radiomic features were extracted representing shape, first-order, gray level co-occurrence matrix−GLCM, gray level run length matrix−GLRLM, gray level size zone matrix−GLSZM, gray level dependence matrix−GLDM, and neighboring gray tone difference matrix−NGTD features at three different levels of granularity (coarse, medium, and fine resampling). Feature extraction was performed using the open-source package pyradiomics v3.0 [32] in compliance with Image Biomarker Standardization Initiative−IBSI standards [33]. As part of our data pre-processing procedure, imaging data were normalized around the mean and standard deviation, and resampling of CT scans to 1, 3, or 5 mm^3^ isotropic voxels was achieved using B-spline interpolation (for fine, medium, and coarse features). Discretization of image intensities was performed within pyradiomics, where we applied different bin widths to capture varying levels of feature granularity: a bin width of 1 for fine features, 5 for medium features, and 25 for coarse features. This approach allowed us to systematically standardize the extraction of texture features across different scales. Detailed information on the feature extraction settings can be found in Supplementary file S1. Collectively, the extracted radiomic features encoded the morphological phenotype of each lesion. An in-depth description of the extracted features can be found in Griethuysen et al [34].

The development of the MSI radiomic predictive signature involved a two-step feature reduction and selection process. Firstly, we used statistical testing to drop features influenced by segmentation and center-level differences. To account for center-level differences, we used the Kruskal-Wallis test to identify features significantly associated with the center of origin. Subsequently, to assess radiomic feature reproducibility due to inter-reader variability, we sampled 36 cases from the training set, and all three radiologists (N.B., F.C., and F.L.) performed segmentations. Features significantly affected by segmentation-level differences, as determined by Mann-Whitney *U* tests, were excluded from the analysis. Benjamini-Hochberg corrections were applied to adjust the significance levels for multiple testing and reduce the false discovery rate. The features deemed robust for center and segmentation were then taken to the second step comprising supervised feature selection using the MSI status outcome.

We used a supervised ensemble feature selection approach where, on the training set, six supervised feature selection techniques (namely, recursive feature extraction, Pearson correlation, χ^2^, logistic regression, light gradient boosting machine, and random forest) were independently tasked with selecting the top 100 features associated with the outcome on the training set. Radiomic features that achieved consensus on relevance (*i.e*., selection by four of six techniques) formed the radiomic signature. Ensemble feature selection methods have been shown to outperform single feature selection methods, generate more stable signatures, and scale to high dimensional data, including radiomics [35–39]. Crucially, this selection step was performed solely on the internal training set, and external validation sets were never used in the second step of the feature selection process (where the MSI status was an input). Additionally, a correlation analysis was performed to ensure that the features in the radiomic signature were sufficiently independent (Fig. 3b).

### Machine learning and modeling

We developed three models tailored to different types of input data: radiomic, clinical, and combined. The radiomic model utilized features from SNUH contrast-enhanced CT scans as both the training and fine-tuning datasets. We employed a five-fold cross-validation method to train and optimize this machine learning (ML) model. For external validation, we combined images from NKI and INT to assess the model’s ability to generalize across various centers and scanning protocols. The clinical and combined models followed the same center-level split strategy for development and validation. Across all models, the distribution of data between the training and external test cohorts adhered to an 80:20 training-test split.

Adaptive synthetic−ADASYN upsampling algorithms were used to address class imbalance between the MSS and MSI patients in the training set. Past radiomic literature has shown that algorithmic upsampling approaches successfully mitigate class imbalance typically encountered in the medical domain [40, 41] and even specifically for radiomic-MSI association [29, 42, 43].

A tree-based evolutionary/genetic algorithm (provided by the Tree-based Pipeline Optimization Tool−TPOT Python library) was used to implement a five-fold cross-validation strategy on the training data. This strategy was applied to both select the optimal ML classifier and fine-tune its hyperparameters [44–46]. Each generation within the evolutionary algorithm consisted of a population of ML classifiers, each with specific hyperparameters. The most successful model/hyperparameter combinations from each generation were selected and carried forward to the next generation. Subsequent generations comprised modified versions (*e.g*., mutations) of these top performers to discover new model/hyperparameter combinations that would enhance predictive performance. Our analysis spanned 100 generations, with a population of 100 models per generation, totaling an exploration of 10,100 ML algorithms before settling on the final model (a decision tree classifier). This final model, emerging from the evolutionary process, underwent testing on the independent external validation data.

### Statistical analysis

Continuous parameters and categorical variables were compared between MSS and MSI patients using the Mann-Whitney *U* and χ^2^ tests, respectively. We assessed inter-rater agreement for radiomic feature extraction using Cohen κ statistic to ensure the reliability of segmentation among different radiologists (using a subset of cases from the training set). Several evaluation metrics were used to measure the predictive performance of the selected ML algorithm on the external validation set. Specifically, we bootstrapped area under the receiving operating curve (AUROC), precision-recall (PR)-AUROC, accuracy, F1 score, sensitivity, specificity, negative predictive value (NPV), and positive predictive value (PPV) 1,000 times to calculate the 95% confidence intervals [CIs]. AUROCs were compared using the approach outlined by Hanley & McNiel [47]. We also computed Shapley values to determine the impact of each feature in the radiomic signature on the prediction made by the ML algorithm. All analyses were conducted using Python (scikit 0.22.1, pandas 1.0.1, numpy 1.18.1, simpleitk 2.3.1, pingouin 0.5.3, matplotlib 3.1.3, tpot 0.11.2, shap 0.37.0).

## Results

### Patient characteristics

This study retrospectively queried baseline data from a total of 367 CRC patients from three independent centers: SNUH (*n* = 231), NKI (*n* = 86), and INT (*n* = 50). MSI status data were unavailable for 69 patients (18.8%), and an additional 47 patients (12.8%) were excluded for radiological reasons (*e.g*., only unenhanced images available, wrong phase, difficult segmentation, etc.). Eight patients (2.2%) were excluded due to technical or image quality reasons. Consequently, the resulting dataset paired MSI status and contrast-enhanced CT scans of 191 SNUH patients, 38 NKI patients, and 14 INT patients. Images from SNUH were assigned to the training/fine-tuning cohort, while scans from NKI and INT scans constituted the external validation cohort. Details of patient inclusion and exclusion are presented in Fig. 1.

The prevalence of MSI was comparable between the training (*n* = 45/191, 23.6%) and the external validation cohort (13/51, 25.5%) cohorts. The median diameter of the segmented lesions was 63.40 mm (IQR 51.1−79.3 mm) with a median volume of 18,436.5 mm^3^ (IQR 9,823.8−30,822.2). Neither diameter (*p* = 0.688) nor tumor volume (*p* = 0.210) was significantly associated with MSI status. Although age was not significantly correlated with MSI status (*p* = 0.172), tumor location (right *versus* left colon) showed a significant association with MSS status (*p* = 0.016). Table 1 provides a detailed breakdown of the patient characteristics.

**Table 1:** Detailed overview of demographic and clinical characteristics of colorectal cancer patients in the study, categorized by participating cohort

	SNUH	NKI	INT
Age (median, IQR)	65.4 (56.8–73.9)	65.8 (60.6–71.1)	61.5 (59.3–75)
Sex (*n*, %)			
Male	98 (51.3%)	20 (52.6%)	6 (42.9)
Female	93 (48.7%)	18 (47.4%)	8 (42.9)
MSI/MSS status (*n*, %)			
MSS	146 (76.4%)	32 (84.2%)	7 (50%)
MSI	45 (23.6%)	6 (15.8%)	7 (50%)
Tumor type			
Right colon	84 (44%)	24 (63.2%)	10 (71.4%)
Left colon	107 (56%)	14 (36.8%)	4 (28.6%)
Maximum three-dimensional diameter, mm (median, IQR)	62.1 (50.1–77.1)	67.8 (56.0–88.1)	67.5 (52.9–80.7)
KRAS status			
Wild type	111 (58.1%)	6 (15.8%)	11 (73.3%)
Mutant	80 (41.9%)	6 (15.8%)	4 (26.7%)
Missing	–	26 (68.4%)	–
BRAF status			
Wild type	98 (51.3%)	9 (23.7%)	9 (60%)
Mutant	8 (4.2%)	5 (13.2%)	6 (40%)
Missing	85 (44.5%)	24 (63.2%)	–

*IQR* Interquartile range, *INT* Istituto Nazionale dei Tumori, *MSI* Microsatellite instability, *MSS* Microsatellite stability, *NKI* Netherlands Cancer Institute, *SNUH* Seoul National University Hospital

### Radiomic signature

For each image, we extracted 6,048 radiomic features that encapsulated the complete morphological phenotype of the primary tumor. Feature reduction/selection was performed via two steps: determination of stable features and supervised ensemble feature selection. Delineations from three radiologists were acquired for a subset of the training set (*n* = 36 patients) to measure variability in segmentation. The inter-rater agreement for these segmentations, showed a median κ value of 0.70 (IQR 0.52–0.79), indicating substantial agreement among the radiologists. Despite this high level of agreement, 320 features were identified as unstable due to inter-reader variability and subsequently excluded from further analysis. We also assessed features for instability related to variations across different centers, resulting in the exclusion of 2,056 features. Consequently, we utilized the remaining 3,887 robust features, which were stable across both segmentation and center-level variations, for the second step of feature reduction/selection.

Ensemble feature selection was implemented on the training data to identify a 7-feature radiomic signature associated with the MSS/MSI status in the SNUH cohort. Each feature of the signature had limited predictive ability when used independently in the unseen external validation dataset (Table 2). Coarse wavelet-LHH_firstorder_Skewness (Feature 2) demonstrated the highest individual discriminatory power (AUROC 0.65). The features in the radiomic signature were largely independent (Fig. 3b) apart from Fine_square_glszm_LargeAreaEmphasis and Fine_square_glszm_ZoneVariance. Both features were retained in the model despite their correlation because each was independently selected by the supervised ensemble feature selection. The full seven-feature signature was used in all subsequent analyses.

**Table 2:** Detailed list of the seven radiomic features comprising the signature for predicting microsatellite instability/stability status in colorectal cancer, along with their individual predictive powers as evaluated on the external validation dataset

Feature name		AUROC value
1	Fine_square_glszm_LargeAreaEmphasis	0.56
2	Coarse_original_firstorder_InterquartileRange	0.52
3	Medium_wavelet-LHH_glszm_LargeAreaLowGrayLevelEmphasis	0.58
4	Medium_square_glszm_LargeAreaLowGrayLevelEmphasis	0.52
5	Fine_square_glszm_ZoneVariance	0.56
6	Coarse_wavelet-LHH_firstorder_Skewness	0.65
7	Coarse_original_glszm_SmallAreaEmphasis	0.52

*AUROC* Area under the receiver operating curve

### Predictive modeling

A tree-based evolutionary algorithm was used to optimize ML algorithms that could predict MSI status (MSS *versus* MSI). Three different models were developed: one using four clinical features often reported as associated with MSI (patient age, tumor side [right *versus* left], diameter, and volume); a second based on the radiomic signature; and a third combining clinical features with the radiomic signature. The algorithm selected a decision tree classifier with optimized hyperparameters as the most suitable model for all three data inputs. The clinical model demonstrated limited predictive capability (average training AUROC 0.61; external validation AUROC 0.54, 95% CI 0.36–0.70, *p* = 0.338). The radiomic model could significantly discriminate between MSS and MSI tumors on the external validation data (average training AUROC 0.71; external validation AUROC 0.69, 95% CI 0.54–0.84, *p* = 0.018). Combining radiomic and clinical features yielded a model with overall improved metrics and a more significant predictive power (average training AUROC 0.76; external validation AUROC 0.78, 95% CI 0.60–0.91, I = 0.002). A detailed comparison of the performance evaluation metrics of the three models is shown in Table 3 and Fig. 3c, d. Supplementary file S2 presents the confusion matrices for the clinical-only, radiomics-only, and combined models, detailing the counts of true positives, false positives, true negatives, and false negatives for each model.

**Table 3:** Effectiveness of the radiomic-only, clinical-only, and combined radiomic-clinical machine learning models in predicting MSI/MSS status, as determined by their performance on the external validation cohort

	Radiomics only	Clinical only	Combined radiomics and clinics
AUROC	0.69 (0.54–0.84)	0.54 (0.36–0.70)	0.78 (0.60–0.91)
PR-AUROC	0.88 (0.78–0.96)	0.76 (0.62–0.90)	0.90 (0.78–0.97)
F1 score	0.76 (0.65–0.87)	0.83 (0.75–0.91)	0.74 (0.61–0.85)
Sensitivity	0.70 (0.55–0.84)	0.95 (0.87–1.00)	0.64 (0.49–0.79)
Specificity	0.62 (0.33–0.89)	0.00 (0.00–0.00)	0.70 (0.43–0.92)
NPV	0.41 (0.18–0.62)	0.00 (0.00–0.00)	0.39 (0.19–0.58)
PPV	0.85 (0.71–0.97)	0.74 (0.62–0.86)	0.87 (0.73–0.97)
Accuracy	0.67 (0.54–0.81)	0.71 (0.60–0.83)	0.65 (0.52–0.79)
*p*-value	0.018	0.34	0.002

*AUROC* Area under the receiver operating curve, *NPV* Negative predictive value, *PPV* Positive predictive value, *PR-AUROC* Precision-recall area under the receiver operating curve

### Model explainability

**Fig. 4:**
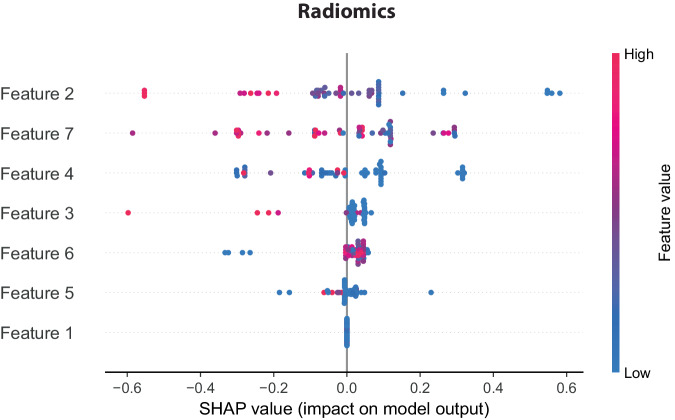
Model interpretability and feature significance. Visualization of feature importance within the machine learning algorithms using Shapley values. These values indicate each feature’s relative contribution to the prediction. The color coding also suggests how higher or lower values of a particular feature influence the model’s prediction (*i.e*., microsatellite instability *versus* microsatellite stability). SHAP, SHapley additive explanation

Shapley values were calculated for each predictive model (radiomics, clinical data, and combined radiomic/clinical data) to highlight the relative importance of the features in the prediction of the algorithm. The SHapley Additive exPlanation−SHAP analysis on the radiomics model indicated that the gray level intensity IQR was the most important feature. The combined radiomics/clinical model used both data types as the basis of its predictions. Figure 4 shows the relative importance of the features within a given model.

## Discussion

This study highlights the potential of radiomic features extracted from routine preoperative CT scans to predict MSI in CRC patients. Our radiomic signature, consisting of seven features, encoded key elements in the morphological phenotype of primary CRC tumors. Individually, the features had limited predictive power, but when combined, they could significantly discriminate between MSS and MSI tumors in an external validation set derived from two independent centers (AUROC 0.69, *p* = 0.018).

Differences in morphology between CRC tumors with MSI and those with MSS are well documented at both the microscopic and macroscopic levels. Histologically, cancers with MSI are often poorly differentiated or mucinous, contain more prominent lymphocytic infiltrate, and show more intratumoral heterogeneity [48, 49]. Similarly, radiological semantic features, including heterogeneity, have previously been associated with MSI/MSS status [50]. Radiomics advances this methodology, offering a less labor-intensive and automated approach to morphological assessment.

In our study, the seven features selected to predict MSI reflected morphological heterogeneity. Among the individual features, skewness demonstrated the highest predictive power (AROUC 0.65). Skewness measures the deviation from a normal signal distribution, suggesting more significant morphological heterogeneity [51, 52]. As a first-order feature, skewness has also been shown to have good reproducibility [53, 54]. In both positron emission tomography/CT and magnetic resonance imaging, skewness was identified as a discriminatory radiomic feature of MSI [55, 56]. Within our algorithm, Shapley value analysis indicated that while skewness was impactful, the algorithm relied more on other radiomic features within the signature (*e.g*., IQR, small area emphasis, and large area low gray level emphasis) to predict MSI status in the external validation set. We believe that the observed shift in reliance from a single feature (*i.e*., skewness) to other radiomic features in the predictive algorithm could be attributed to the complex and multifaceted nature of MSI, which likely cannot be fully characterized by a single parameter alone. While skewness offers valuable insights into distribution asymmetry, it may only capture some nuanced texture or spatial data essential for precise predictions. Other features, such as the IQR, small area emphasis, and large area low gray level emphasis, provide complementary information on morphological heterogeneity, enhancing the model’s predictive power.

The patient characteristics in both our training and validation sets aligned with the general CRC population, showing a higher prevalence of MSI in the right colon, as reported in previous studies [57]. Age and tumor location are often cited as highly linked with MSI [4]. Although the tumor location was found to be associated with MSI status in our study (*p* = 0.016), it was not a strong enough predictor on its own. A model trained on clinical parameters alone showed no significant predictive capacity.

Integrating clinical features with our radiomic signature significantly enhanced the predictive model (AUROC 0.78, *p* = 0.002), surpassing the performance of the radiomics-only model (AUROC 0.69, *p* = 0.018). This improvement may be attributed to the complementary information that clinical features provide, capturing aspects of the tumor characteristics that are not fully reflected in radiomic features/morphology alone. Combining these two data sources effectively enriches the model’s predictive capacity, allowing for a more comprehensive and nuanced understanding of the complexities inherent in MSI. Consistent with our findings, two monocentric studies have demonstrated the enhanced prediction of MSI status achieved by combining radiomic and clinical features [58, 59]. Ying et al trained a model on a similarly sized dataset to develop a rad-score model that scored MSI patients higher (AUROC 0.89). When exploring clinical parameters, they included both routine clinical parameters (*e.g*., location of the primary tumor, white blood cell status, histological grade) and semantic features (*e.g*., CT-reported inflammatory response) into a nomogram with the rad-score [58]. As in our study, the model’s performance improved by implementing potentially complementary data (AUROC 0.90). In our study, we did not explore the potential added value of semantic features; however, such features could enhance predictive performance.

Yuan et al [59] notably combined tumoral and peritumoral radiomic features from patients with rectal cancer. Their model’s performance showed similar levels of predictive power as our radiomics model using features from the tumor alone. The combined radiomic-clinicopathological nomogram performed better (AUROC 0.737) than radiomics alone (AUROC 0.726) [59]. While this study only shows a small performance increase (without statistical testing), overall the concept of integrating different types of diagnostic data promises to provide machine learning models with more complementary data to learn.

Single-center studies have explored the use of imaging markers to differentiate MSI/MSS CRC tumors in CT, magnetic resonance imaging, and positron emission tomography/CT [4, 29, 42, 55, 56, 58–61]. However, external validation of MSI radiomic literature is very scarce, with only one other study using conventional CT radiomics and another two using magnetic resonance imaging radiomics [43, 62, 63]. Cao et al identified a 32-feature signature derived from enhanced triphasic CT scans capable of predicting MSI [64].

Although our study has shown promising results, we must acknowledge certain limitations. The retrospective nature of our research, coupled with a modest cohort size, may impact the generalizability of our findings. While the radiomic signature identified and predictive algorithm have shown encouraging results on an external validation cohort from two independent centers, we recognize that radiomic features are susceptible to many factors (*e.g*., scanner models, acquisition protocol, and feature definition differences) [65–67]. In our feature selection/reduction process, we dropped features that were not robust for center-level differences. This step, while critical to enhancing the robustness of our model, used data from both the training and testing sets to determine feature stability across centers, inadvertently introducing a potential source of bias. Although this step did not directly involve class labels and thus did not leak specific MSI information, it may have influenced feature selection, possibly affecting the model’s ability to generalize. Machine learning models trained on larger heterogeneous training datasets from multiple centers could have even greater robustness and reproducibility.

Despite the moderate sensitivity (0.64) and relatively low NPV (0.39) of our combined clinical-radiomics approach, we believe that future implementation of such an algorithm into clinical routines would offer several potential benefits that complement traditional biopsy-based diagnostics. Firstly, artificial intelligence models could serve as preliminary non-invasive screening tools to help prioritize patients for further genetic testing. This would be particularly impactful in clinical settings where resources are constrained (in peripheral hospitals or in developing economies) or where invasive procedures pose substantial risks. Noninvasive radiomic approaches might be helpful in screening for Lynch syndrome or MSI across different cancer types, where MSI status can influence treatment decisions, especially after the United States Food and Drug Administration approval of pembrolizumab for all MSI-high tumors [28]. By potentially identifying MSI cases from routine imaging before a biopsy, the model facilitates early planning and personalized treatment strategies.

In cases where the radiomics model suggests MSI presence, clinicians can expedite confirmatory testing and tailor treatment plans more swiftly. Conversely, while patients identified by the model as MSS might still require confirmatory biopsies, the model helps to reduce the urgency or frequency of invasive tests in low-risk patients, thereby streamlining the diagnostic process. In addition, the model’s capacity to integrate with existing clinical workflows—utilizing readily available preoperative CT scans—enhances its practical utility without requiring additional procedures or costs.

Acknowledging the limitations of our model’s current metrics, ongoing efforts to refine the radiomic signature by incorporating a broader range of imaging (radiomics and nonradiomic) and clinical features are expected to improve its accuracy. For instance, semantic features regarding the morphology of a lesion could be advantageous in lesion types challenging to delineate (*e.g*., ulcerative or depressed lesions). As radiomic technology evolves and more data becomes available, continuous learning models could dynamically update and enhance their predictive accuracy. Therefore, while our model may not replace histopathological testing (nor does it aim to), it serves as an indication that radiogenomic approaches for MSI prediction can augment the diagnostic toolkit, offering a valuable preliminary assessment tool that can guide more focused and efficient use of medical resources in the management of CRC as well as other tumor types.

In summary, radiomic signatures encoding the tumor morphological phenotype predicted the MSI status in CRC. We successfully validated a predictive algorithm on retrospective data from two independent external validation centers. Combining radiomic and clinical parameters improved the predictive performance of the models, suggesting the promise of integrating complementary diagnostic data. Larger-scale multicenter training and prospective validations are needed to bring this radiogenomic approach closer to clinical implementation.

### Supplementary information

**Additional file 1: Supplementary S1. Radiomic feature extraction.** Handcrafted radiomic features (n = 6,048) were extracted from each three-dimensional segmented tumour using pyradiomics (v3.0). The parameters below were used as the settings of the feature extraction on pyradiomics. The heading “ImageType” defines the filters applied to the different classes of radiomic features in “featureClass.” Further details on the precise definition of parameters can be found in the pyradiomics documentation. **Supplementary S2.** Confusion matrix comparison for the different models outlined in this study.

## Data Availability

The datasets generated and/or analyzed during the current study are not publicly available due to institutional and national patient privacy regulations but are available from the corresponding author upon reasonable request.

## Abbreviations

AUROC: Area under the receiving operating curve
CI: Confidence interval
CRC: Colorectal cancer
CT: Computed tomography
INT: Fondazione IRCCS Istituto Nazionale dei Tumori
IQR: Interquartile range
ML: Machine learning
MMR: Mismatch repair
MSI: Microsatellite instability
MSS: Microsatellite stability
NKI: Netherlands Cancer Institute
NPV: Negative predictive value
PPV: Positive predictive value
PR-AUROC: Precision-recall AUROC
SNUH: Seoul National University Hospital
