# Supplementary material for: Non-invasive CT radiomic biomarkers predict microsatellite stability status in colorectal cancer: a multicenter validation study
Source: Eur Radiol Exp. 2024 Aug 26;8:98. doi: 10.1186/s41747-024-00484-8 (PMC11347521; doi:10.1186/s41747-024-00484-8)
Supplement: Supplementary file 1 — Additional file 1: Supplementary S1. Radiomic feature extraction. Handcrafted radiomic features (n = 6,048) were extracted from each three-dimensional segmented tumour using pyradiomics (v3.0). The parameters below were used as the settings of the feature extraction on pyradiomics. The heading “ImageType” defines the filters applied to the different classes of radiomic features in “featureClass.” Further details on the precise definition of parameters can be found in the pyradiomics documentation. Supplementary S2. Confusion matrix comparison for the different models outlined in this study. [file 41747_2024_484_MOESM1_ESM.pdf]

Non-invasive CT radiomic biomarkers predict microsatellite stability status in colorectal cancer: a multicenter validation study

ELECTRONIC SUPPLEMENTARY MATERIAL

Supplementary S1. Radiomic feature extraction. Handcrafted radiomic features (n=6048) were extracted from each three-dimensional segmented tumour using pyradiomics (v3.0). The parameters below were used as the settings of the feature extraction on pyradiomics. The heading "ImageType" defines the filters applied to the different classes of radiomic features in "featureClass." Further details on the precise definition of parameters can be found in the pyradiomics documentation.

```
imageType:
  Original: {}
  Square: {}
  SquareRoot: {}
  Logarithm: {}
  Exponential: {}
  Gradient: {}
  LoG:
    sigma: [1.0, 2.0, 3.0, 4.0, 5.0]
  Wavelet: {}
  LBP3D:
    binWidth: 1.0


featureClass:
  shape:
  firstorder:
  glcm:
    - 'Autocorrelation'
```

  - 'JointAverage'

  - 'ClusterProminence'

  - 'ClusterShade'

  - 'ClusterTendency'

  - 'Contrast'

  - 'Correlation'

  - 'DifferenceAverage'

  - 'DifferenceEntropy'

  - 'DifferenceVariance'

  - 'JointEnergy'

  - 'JointEntropy'

  - 'Imc1'

  - 'Imc2'

  - 'Idm'

  - 'Idmn'

  - 'Id'

  - 'Idn'

  - 'InverseVariance'

  - 'MaximumProbability'

  - 'SumEntropy'

  - 'SumSquares'

 glrlm:

 glszm:

 gldm:

```
  ngtdm:

setting:
  interpolator: 'sitkBSpline'
  resampledPixelSpacing: [1, 1, 1] ([3, 3, 3] for medium; [5, 5, 5] for coarse features)
  padDistance: 10
  minimumROIDimensions: 2
  minimumROISize: 1
  correctMask: true
  binWidth: 1 (5 for medium; 25 for coarse features)

  voxelArrayShift: 1000

  label: 1
```

Supplementary S2. Confusion matrix comparison for the different models outlined in this study.

Model Name	True positive	False positive	True Negative	False Negative
Clinical-only model	38	12	1	1
Radiomics-only model	27	7	6	12
Combined model	33	7	6	6
